# Scoliosis Associated with Lumbar Spondylolisthesis: Spontaneous Resolution and Seven-Year Follow-Up

**DOI:** 10.7759/cureus.6904

**Published:** 2020-02-06

**Authors:** ‏Mohammed ‏Khashab, Bandar N AlMaeen, Mohamed Elkhalifa

**Affiliations:** 1 ‏Orthopedic Surgery, King Abdulaziz Medical City / Ministry of National Guard - Health Affairs, Jeddah, SAU; 2 Orthopedic Surgery, College of Medicine, AlJouf University, AlJouf, SAU; 3 Orthopedic Surgery, King Abdulaziz Medical City / Ministry of National Guard - Health Affairs, Jeddah, SAU

**Keywords:** scoliosis, mohammed khashab, bandar almaeen, mohamed elkhalifa, spontaneous resolution of scoliosis, spondylolisthesis, kind abdulaziz medical city, aljouf university, jeddah

## Abstract

Scoliosis is defined as a structural deformity of the spine in all three dimensions and diagnosed if the Cobb angle is ≥10 degrees. Scoliosis is frequently associated with symptomatic spondylolisthesis, with an incidence ranging from 15% to 48%. The present report describes a patient with scoliosis associated with grade IV lumbar dysplastic spondylolisthesis who experienced the spontaneous correction of scoliosis after spondylolisthesis correction and fixation. The patient was a 12-year-old girl premenarche with an eight-month history of progressively increasing scoliosis, including back pain, left side leg pain, spinal deformity, and abnormal gait. She had been treated with a brace at the referring hospital but without significant improvement. Anteroposterior radiographs showed a long section of the spine, from T2 to L2, curving about 28.8 degrees to her right side, without evident pedicle rotation. Lateral radiographs revealed L5/S1 dysplastic type spondylolisthesis with >75% slippage (Meyerding Grade IV), a dome-shaped sacrum, and a flat back with butterfly sign. Correction of her spondylolisthesis by segmental instrumentation and interbody fusion of L5 and S1 resulted in almost complete resolution of her pain and scoliosis, with the outcome remaining stable seven years after surgery. These findings indicate that patients with scoliosis caused by spondylolisthesis may require only surgery for the latter condition, avoiding unnecessary surgery for scoliosis.

## Introduction

Scoliosis is defined as a structural deformity of the spine in all three dimensions, coronal, sagittal, and axial. This condition is assessed by measuring the major curves comprising the deformity using the Cobb method, with scoliosis diagnosed as a Cobb angle ≥10 degrees. In addition to spinal curves, scoliosis is usually associated with asymmetries of the trunk and the extremities [1]. Scoliosis is also frequently associated with spondylolisthesis, with an incidence ranging from 15% to 48%, and is believed to be more common in patients with spondylolisthesis at the L4-L5 level and those with dysplastic spondylolisthesis [2-5]. In the latter group, the incidence of scoliosis was found to be associated with the degree of slip, particularly when this exceeded 25%. The incidence of scoliosis was low, however, in patients with isthmic spondylolisthesis at the lumbosacral junction. Most of the curves are lumbar, with the thoracolumbar curve usually not exceeding 15 degrees [6].

The frequent association between spinal curves and symptomatic spondylolisthesis indicates that the management of patients with both conditions must consider the entire spinal deformity. The role of surgery in treating patients with spondylolisthesis and scoliosis has not been well defined. This report describes a young girl with symptomatic spondylolisthesis complicated by progressive scoliosis who benefited from the surgical treatment of spondylolisthesis alone.

## Case presentation

A 12-year-old girl premenarche, with an eight-month history of progressively increasing scoliosis, reporting concerns of back pain, left side leg pain, spinal deformity, and abnormal gait was referred to our hospital for surgical correction of scoliosis. She had been treated with a brace at the referring hospital but without significant improvement. Except for scoliosis, she was otherwise healthy. Physical examination revealed an abnormal posture, and an Adam forward bending test showed a rotation of the thoracic spine by about 10 degrees, with an uncompensated gait and a trunk shift toward the left side. Her right shoulder was higher than her left shoulder, with flat back syndrome and she had generalized lumbar spine tenderness mainly at the lumbosacral junction. She was positive for straight leg raising on the left side and showed left side lower limb ankle plantar flexion weakness collectively 3/5, with her ankle reflex being depressed compared with the contralateral side. She also experienced reduced sensations in her left leg and foot, but no changes in the skin.

**Figure 1:**
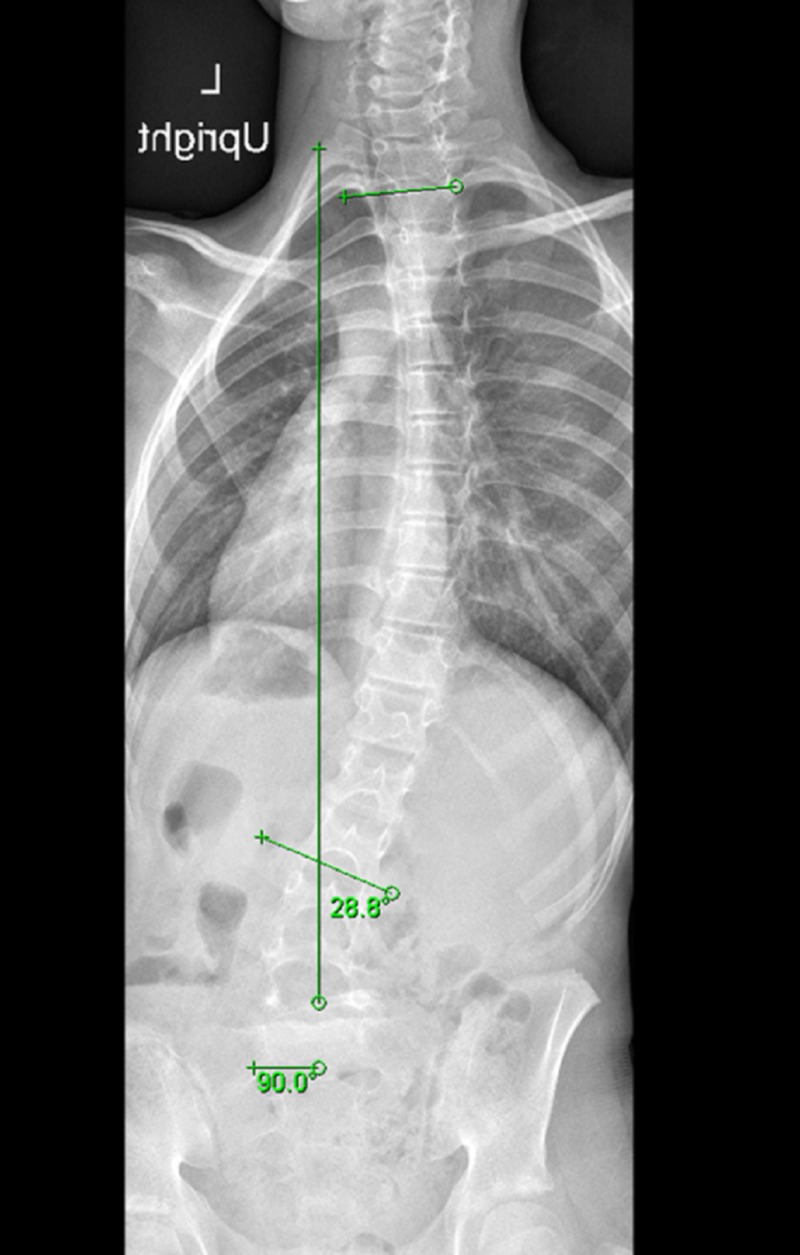
Weight-bearing anteroposterior full spine scoliosis radiograph showing a long section curve of the spine to the right side about 28.8 degrees starting from T2 to L2 without evident pedicle rotation

**Figure 2:**
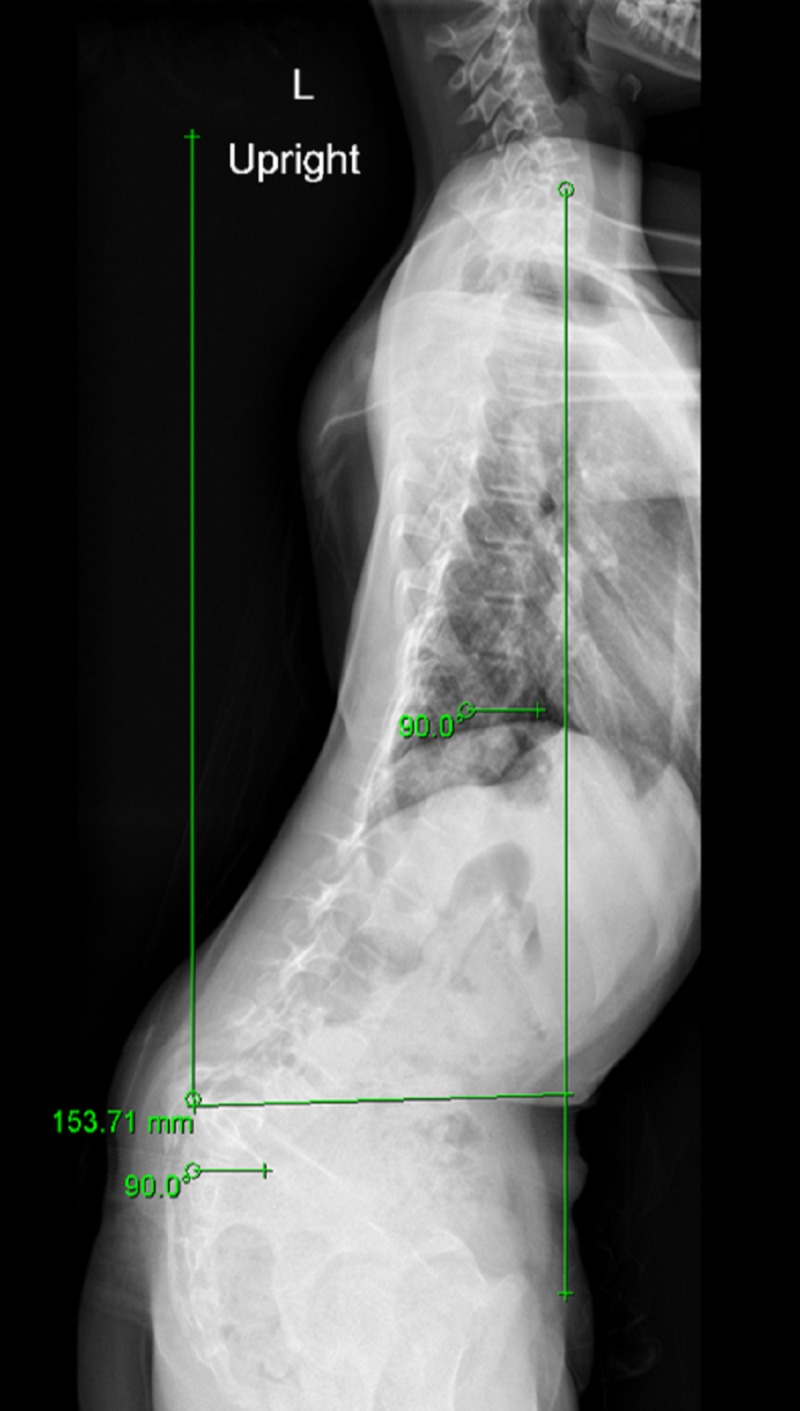
Lateral radiograph reveals L5/S1 dysplastic type spondylolisthesis with more than 75% slippage (Meyerding Grade IV), a dome-shaped sacrum, and flat back with butterfly sign

**Figure 3:**
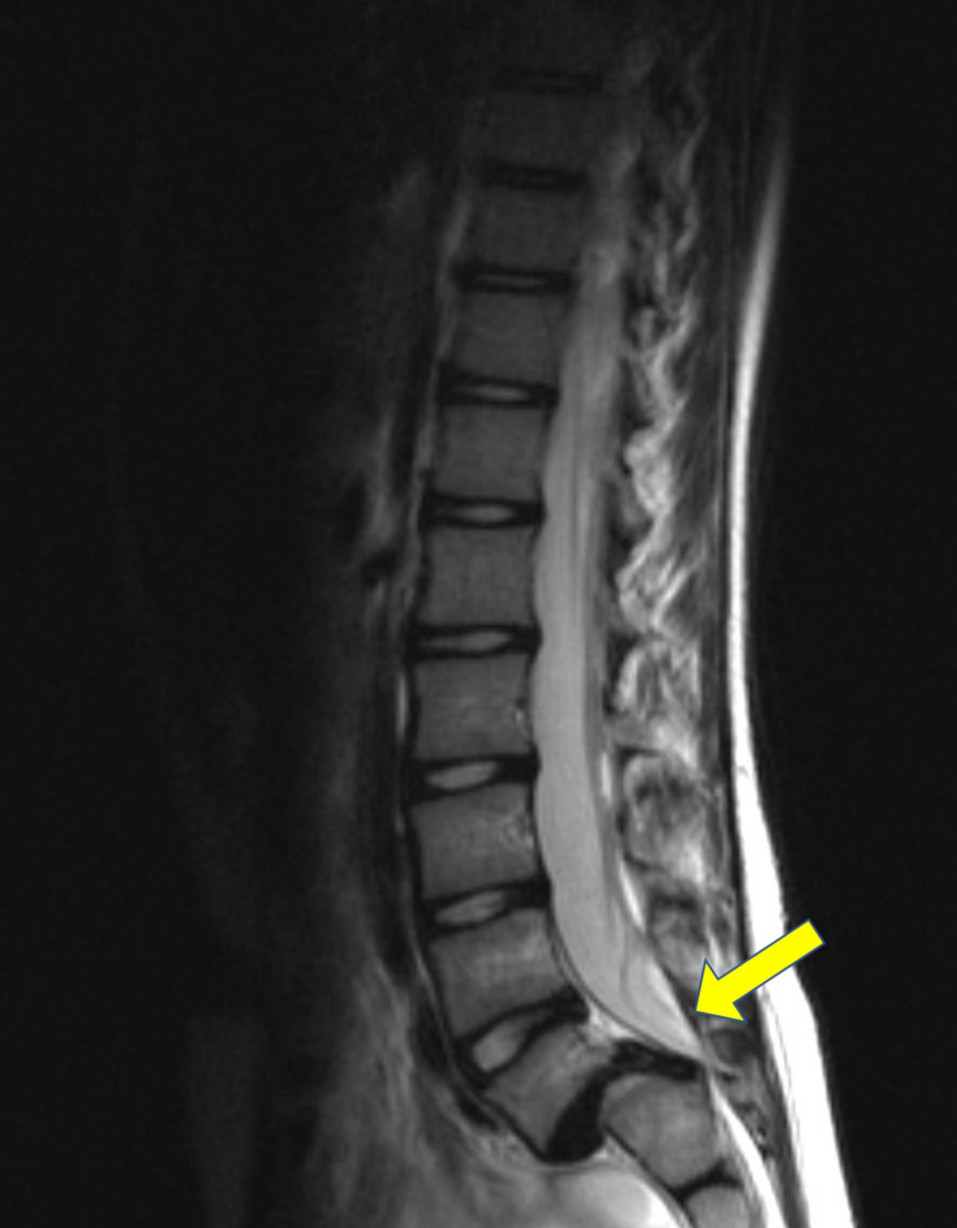
MRI image shows Grade IV spondylolisthesis associated with a large diffuse disk bulge at L5-S1 level causing indentation of the ventral aspect of the thecal sac MRI, magnetic resonance imaging.

**Figure 4:**
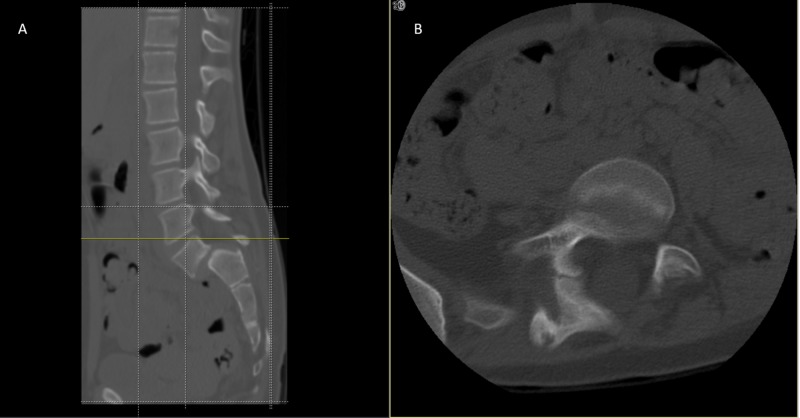
CT scan (A) sagittal and (B) axial plane image showed a very large lamina defect at the level of L4-L5 presenting spina bifida, L5 vertebral body wedging and irregularity of the inferior endplate, L5 bilateral pars interarticularis defect with Grade IV spondylolisthesis CT, computed tomography.

**Figure 5:**
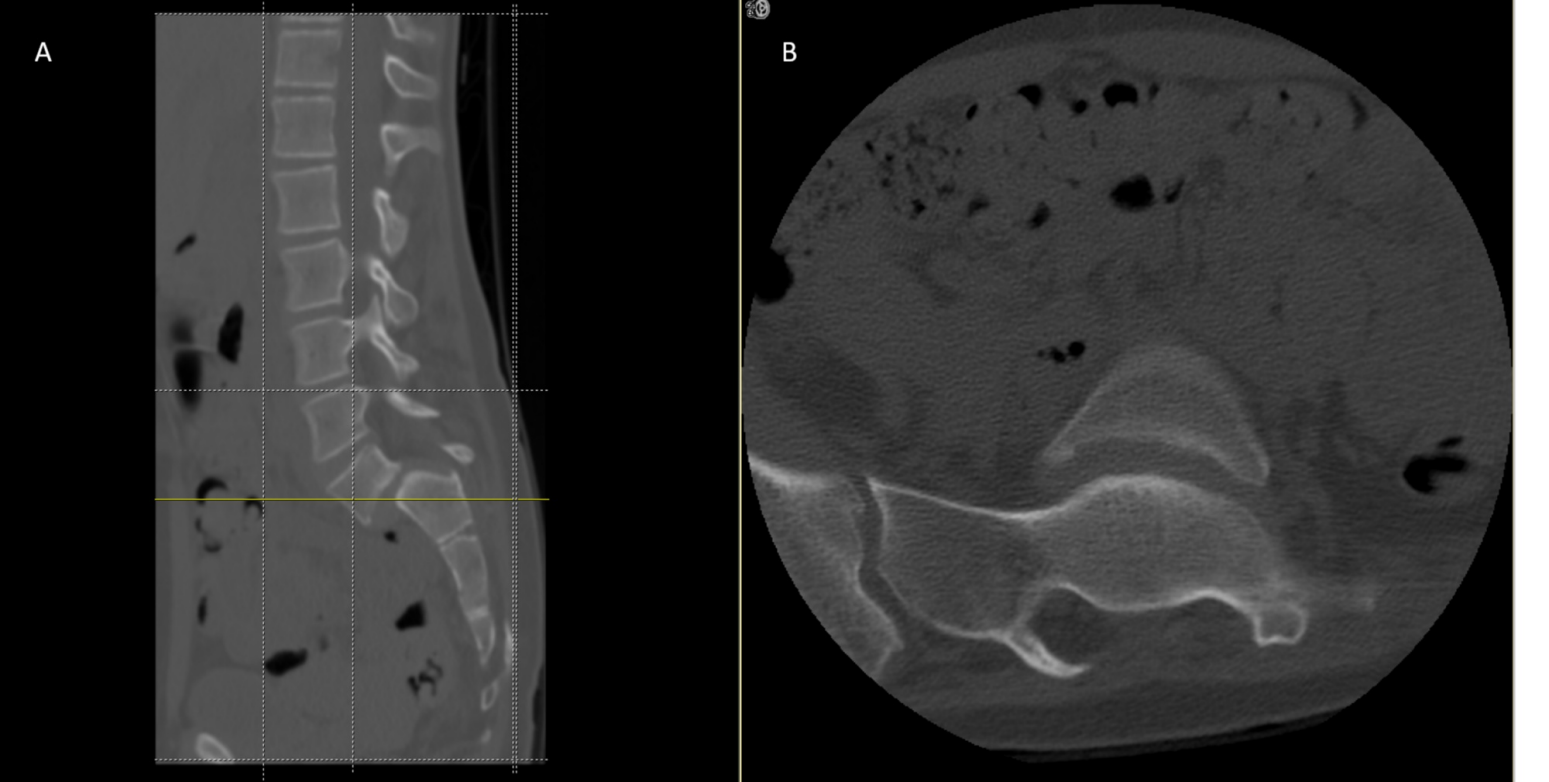
CT scan (A) sagittal and (B) axial plane image showing a very large lamina defect at the level of S1-S2 presenting spina bifida, L5 vertebral body wedging and irregularity of the inferior endplate with Grade IV spondylolisthesis CT, computed tomography.

Weight-bearing anteroposterior full spine scoliosis radiographs were obtained and showed a long section curve of the spine, starting from T2 to L2, about 28.8 degrees to her right side, without evident pedicle rotation (Figure 1). Lateral radiographs revealed L5/S1 dysplastic type spondylolisthesis with >75% slippage (Meyerding Grade IV), a dome-shaped sacrum, and a flat back with butterfly sign (Figure 2). Computed tomography and magnetic resonance imaging showed a scoliotic S-shaped deformity, along with a very large lamina defect extending from L5 to S4 presenting as spina bifida; L5 vertebral body wedging and irregularity of the inferior endplate. An L5 bilateral pars interarticularis defect was observed, along with grade IV spondylolisthesis associated with a large diffuse disk bulge at the L5-S1 level, causing indentation of the ventral aspect of the thecal sac associated with narrowing of the bilateral neural foramina (Figure 3, Figure 4, Figure 5).

**Figure 6:**
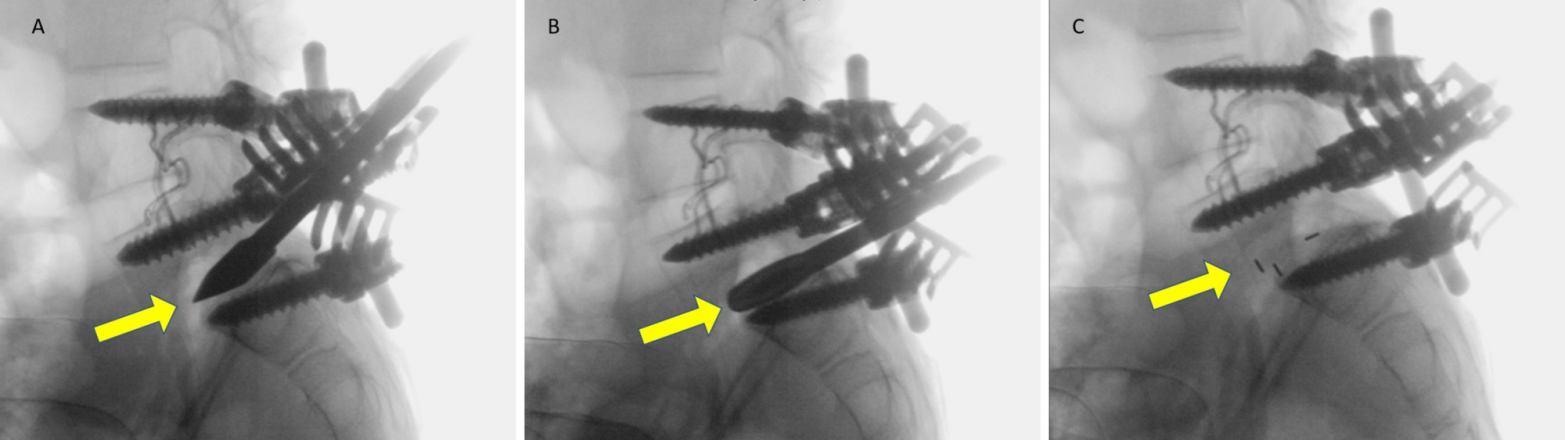
Intraoperative fluoroscopic lateral radiographs show (A) L5 reduction and S1 dome-shaped vertebra post osteotomy, posterior decompression, and segmental posterior instrumentation L4 to S1 (B). (C) shows the circumferential fusion of L5 and S1 by transforaminal lumbar interbody fusion technique

The patient was admitted and prepared for surgery in which the spondylolisthesis was treated, and the indication for the surgery was our patient is a growing child with asymptomatic L5/S1 spondylolisthesis, slippage >75%, an uncompensated gait, and a high risk of further progression. Surgery for spondylolisthesis under neuromonitoring (real-time) was uneventful, with blood loss estimated as 500 cc. The operation included posterior decompression, segmental posterior instrumentation from L4 to S1 with intraoperative reduction of L5, and circumferential fusion of L5 and S1 by transforaminal lumbar interbody fusion technique (Figure 6A, 6B, 6C). Spina bifida was observed, with exposed dura on the corresponding level. There were no surgical or postoperative complications.

After surgery, the patient was transferred to the pediatric intensive care unit for 24 hours and then to a regular ward. She was in good condition and started mobilization immediately with a walking frame and the support of a physiotherapist. Within a few days the patient tolerated the postoperative period, she was mobilizing alone, and her wound was fine. She was discharged home on day seven in good condition and prescribed pain medications only.

**Figure 7:**
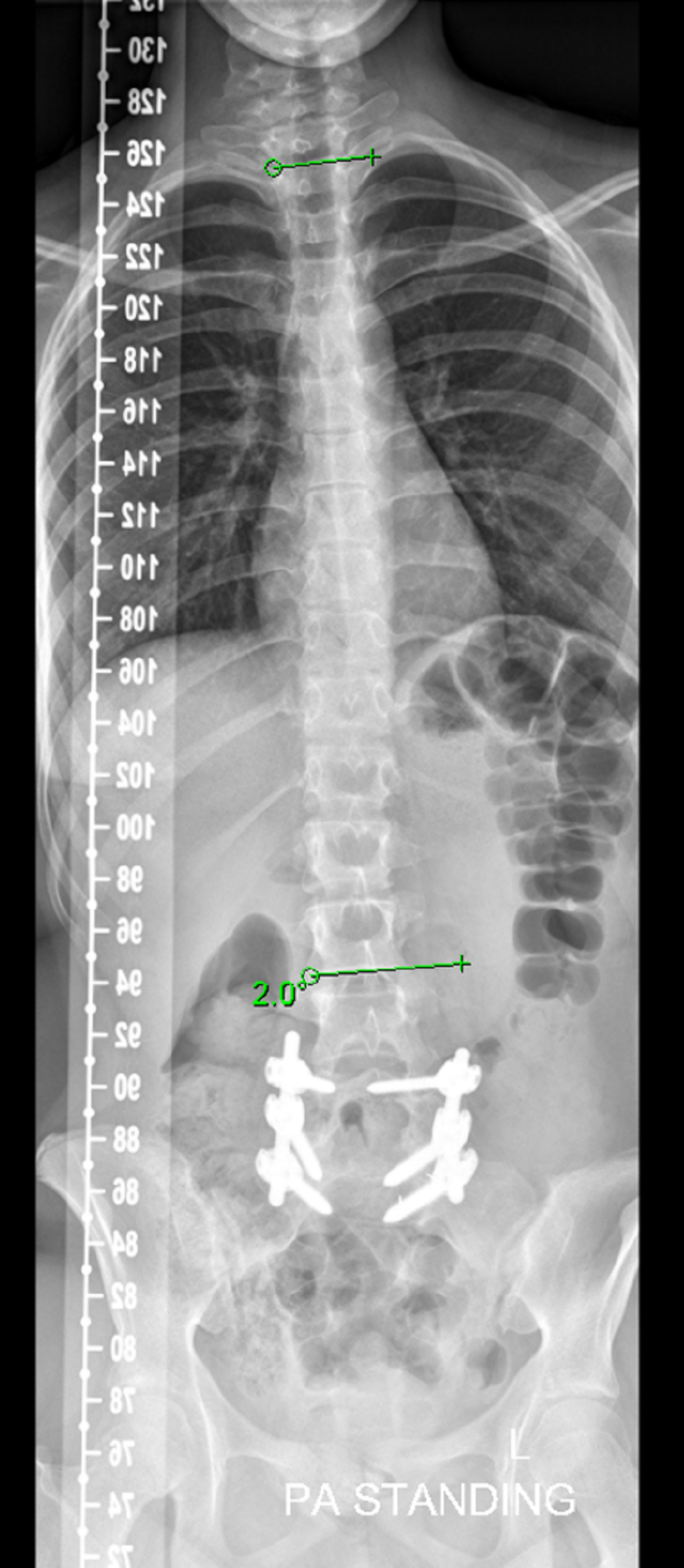
Follow-up weight-bearing anteroposterior full spine Scoliosis radiograph showing a well-balanced alignment of the spine with correction of her scoliotic curve

**Figure 8:**
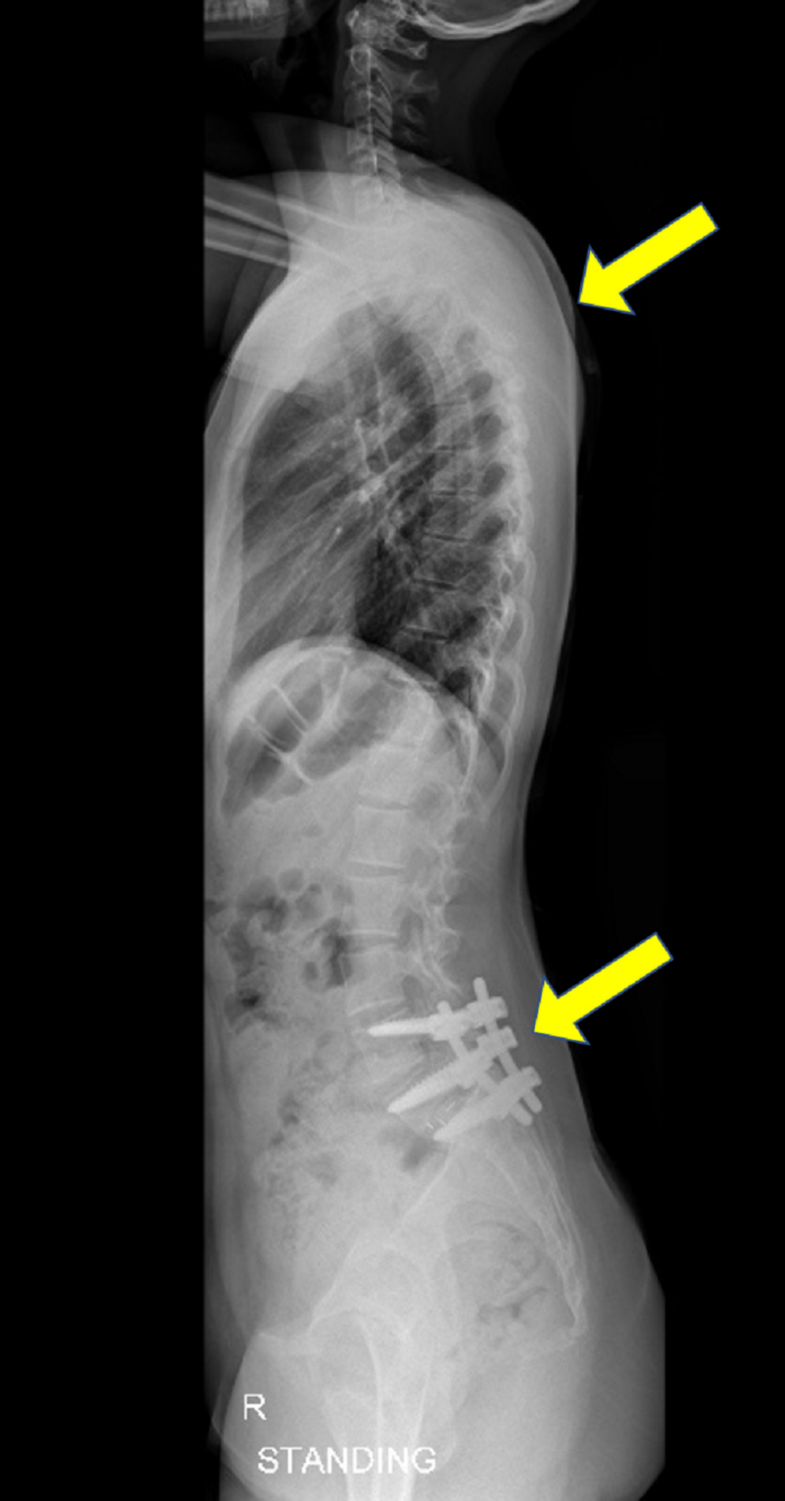
Follow-up lateral full spine scoliosis radiograph showing well-balanced alignment of the spine with correction of her scoliotic curve

The patient was followed up one, two, five, seven, and 12 months after surgery, once every six months and annually thereafter. CT showed complete reduction and fusion of L5-S1 spondylolisthesis immediately after surgery and during follow-up. During this time, her scoliosis showed spontaneous resolution and was completely corrected (Figure 7, Figure 8), and she was able to return to her previous activities. Follow-up examinations of this patient over seven years have shown continued excellent relief of her symptoms, with a well-balanced posture and no gross deformity. Radiographs showed a well-balanced alignment of the spine with the correction of her scoliotic curve (Figure 7, Figure 8).

## Discussion

Three types of scoliotic curve patterns have been associated with spondylolisthesis. The first type is idiopathic scoliosis, which frequently involves the upper, thoracic, or thoracolumbar spine. The second type is olisthetic or torsional scoliosis, which can be a result of asymmetric rotation and displacement from the spondylolytic defect. The third type is sciatic or spondylolytic, also called antalgic, scoliosis, which can result from sciatic irritation or muscle spasms. Compensatory olisthetic and sciatic scoliosis tend to resolve spontaneously if lumbosacral fusion is performed before the curve becomes structural [2-11].

A study of 78 patients with scoliosis and spondylolisthesis treated over 14 years found that scoliosis severe enough to be treated should be regarded as idiopathic, with scoliosis treated separately from spondylolisthesis, if the latter is asymptomatic. If spondylolisthesis is symptomatic, however, both conditions should be treated. Depending on the severity of the curve, scoliosis and spondylolisthesis should be treated at the same time, or else scoliosis should be treated after spondylolisthesis, with curves that are more scoliotic being treated surgically or conservatively [12].

Patients who present with spondylolisthesis and evidence of scoliosis should undergo an evaluation of the entire spine. This evaluation is necessary to identify the type of curve and its relationship to the neural arch defect. Furthermore, it is very important to identify any structural deformities as well as whether the curve is fixed or flexible before choosing the appropriate treatment plan [13].

**Figure 9:**
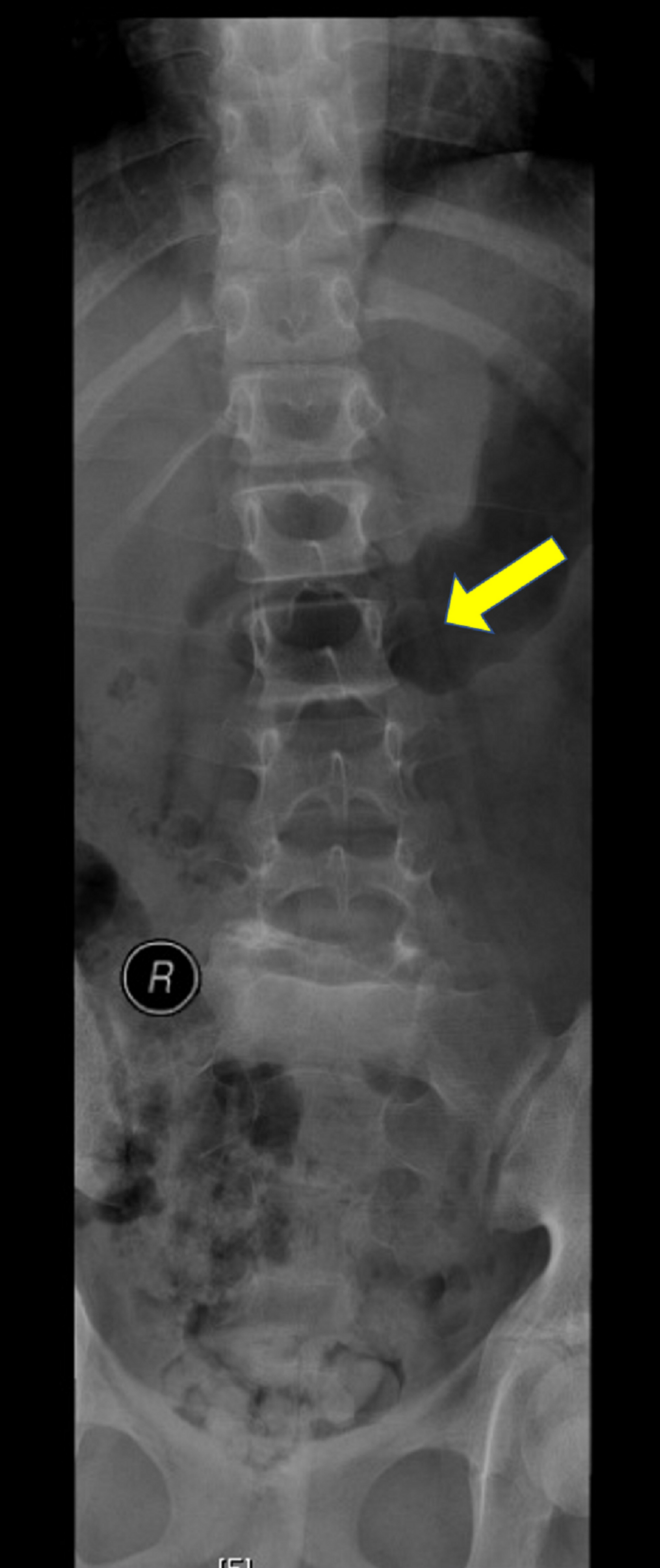
Preoperative radiographs demonstrating the absence of vertebral body rotation and correction of the scoliotic curve in the supine position

Patients who present with both idiopathic scoliosis and spondylolisthesis unrelated to each other require separate treatments of both lesions. Scoliotic curves resulting from sciatic irritation secondary to a slip, but not yet structural or fixed, as shown by overcorrection on lateral bending radiographs, the absence of vertebral body rotation and correction of the curve in the supine position, as in our patient (Figure 9), are thought to correct spontaneously after lumbosacral fusion. This will eliminate the cause of the spasm, resulting in the correction of the curve in most patients. Curve correction may be prevented by the occurrence of structural changes or the development of a fixed contracture on the concave side due to long-standing spasms. Early treatment of these patients is recommended before the deformity becomes fixed. Olisthetic and torsional curves also respond well to lumbosacral fusion [2-3,6,9].

The present patient presented with symptoms of both scoliosis and spondylolisthesis, including recent onset of low back pain, left side leg pain, spinal deformity, and abnormal gait with progressive scoliosis. The absence of vertebral body rotation associated with a >75% slip of L5 on S1 indicated that this patient had a nonstructural and flexible sciatic curve. Surgery is indicated for patients who experience curve progression despite nonoperative management and those with curves >50%; thus, the spinal curvature and symptoms in our patient were severe enough to warrant surgical management. Although guidelines suggest that spondylolisthesis and scoliosis in our patient should be treated simultaneously, her scoliosis and symptoms resolved spontaneously and completely after lumbosacral fusion surgery for spondylolisthesis. Unnecessary surgery for scoliosis was avoided, with the results showing that the treatment protocol was effective and less invasive. There is a complex relationship between scoliosis and spondylolisthesis. Greater knowledge about the etiology and manifestations of scoliosis associated with spondylolisthesis may help in the future treatment of patients with these two spinal disorders.

## Conclusions

The relationship between scoliosis and spondylolisthesis is complex. If scoliosis is regarded as caused by spondylolisthesis, surgery for the latter condition may be the only required intervention, avoiding the need for unnecessary surgery for scoliosis.

## References

[1] Cheng JC, Castelein RM, Chu WC (2015). Adolescent idiopathic scoliosis. Nat Rev Dis Primers.

[2] Seitsalo S, Osterman K, Poussa M (1988). Scoliosis associated with lumbar spondylolisthesis. A clinical survey of 190 young patients. Spine.

[3] Fisk JR, Moe JH, Winter RB (1978). Scoliosis, spondylolysis and spondylolisthesis. Their relationship as reviewed in 539 patients. Spine.

[4] Arlet V, Rigault P, Padovani JP, Touzet P, Finidori G, Guyonvarch G (1990). Scoliosis, spondylolysis and lumbosacral spondylolisthesis. A study of their association apropos of 82 cases in children and adolescents. Rev Chir Orthop Reparatrice Appar Mot.

[5] Libson E, Bloom RA, Shapiro Y (1984). Scoliosis in young men with spondylolysis or spondylolisthesis. A comparative study in symptomatic and asymptomatic subjects. Spine.

[6] McPhee IB, O’Brien JP (1980). Scoliosis in symptomatic spondylolisthesis. J Bone Joint Surg Br.

[7] Tojner H (1963). Olisthetic scoliosis. Acta Orthop Scand.

[8] Mau H (1981). Scoliosis and spondylolysis-spondylolisthesis. Acta Orthop Trauma Surg.

[9] Goldstein LA, Haake PW, Devanny JR, Chan DPK (1976). Guidelines for the management of lumbosacral spondylolisthesis associated with scoliosis. Clin Orthop Relat Res.

[10] Risser JC, Norquist DM (1961). Sciatic scoliosis in growing children. Clin Orthop.

[11] Peterson JB, Wenger DR (2008). Asymmetric spondylolisthesis as the cause of childhood lumbar scoliosis—can new imaging modalities help clarify the relationship?. Iowa Orthop J.

[12] Crostelli M, Mazza O (2013). AIS and spondylolisthesis. Eur Spine J.

[13] Pneumaticos SG, Esses SI (2003). Scoliosis associated with lumbar spondylolisthesis: a case presentation and review of the literature. Spine J.

